# Clinical features of adolescents diagnosed with eating disorders and at risk for psychosis

**DOI:** 10.1192/j.eurpsy.2020.80

**Published:** 2020-08-24

**Authors:** Martina Maria Mensi, Chiara Rogantini, Renata Nacinovich, Anna Riva, Livio Provenzi, Matteo Chiappedi, Umberto Balottin, Renato Borgatti

**Affiliations:** 1 Child Neurology and Psychiatry Unit, IRCCS Mondino Foundation, Pavia, Italy; 2 Child Neurology and Psychiatry Unit, Brain and Behavioral Sciences Department, University of Pavia, Pavia, Italy; 3 Child and Adolescent Mental Health, San Gerardo Hospital, Monza, Italy; 4 School of Medicine and Surgery and Milan Center for Neuroscience, University of Milano Bicocca, Milano, Italy

**Keywords:** Adolescence, anorexia, eating disorder, psychosis

## Abstract

**Background.:**

The presence of subthreshold psychotic symptoms in adolescents with eating disorders is poorly described. This study provides a detailed characterization of adolescents affected by eating disorders in the absence or presence of subthreshold psychotic symptoms, taking into account a wide set of sociodemographic, psychological, and clinical variables.

**Methods.:**

Ninety-four adolescents diagnosed with eating disorders were interviewed, focusing on clinical anamnesis and sociodemographic data collection. The Comprehensive Assessment of At-Risk Mental States (CAARMS) was used to assess the presence (HR+) or absence (HR−) of subthreshold psychosis. The clinicians completed a questionnaire on eating disorders severity, whereas patients provided self-report measures of global social functioning and psychological symptoms associated with eating disorders.

**Results.:**

Attenuated psychotic symptoms were highly frequent (84% of subjects). HR+ patients experienced more frequently purging behaviors and dysmorphophobia and received a greater amount of antipsychotic drugs. Compared to HR− counterparts, HR+ patients reported higher eating disorders severity and psychological symptoms (i.e., ineffectiveness, interpersonal and affective problems) associated with eating disorders. Finally, a significant correlation between global social functioning and eating disorders severity emerged only for HR− subjects.

**Conclusions.:**

These descriptive data are warranted to identify a potential psychotic core in eating disorders, mainly concerning body image and weight as well as specific psychological features. The availability of reliable and valid markers of risk can further increase our capacity to detect the early emergence of psychosis in adolescents with eating disorders, whose outcome might be worsened by the presence of psychotic symptoms.

## Introduction

Eating disorders are a heterogeneous group of potentially severe and often chronic pathologies, accompanied by an important impairment of global, social, and labor functioning [1]. They are among the most common chronic mental illnesses in adulthood, but they show a high incidence rate and a peak onset in adolescence, between the ages of 14 and 19 [2]. The healthcare journey of these patients is usually prolonged and requires a multispecialist care team. In addition, they are associated with up to 9.4% rate of mortality (1.8% in adolescents) after more than 10 years of follow-up, with a fifth of them being suicidal deaths [3].

It is widely known that eating disorders are frequently comorbid with other psychiatric disorders, with a lifetime prevalence, in subjects with anorexia nervosa, between 45 and 97% [2]. The presence of other psychiatric disorders usually correlates with less favorable outcomes, exacerbating the severity of eating disorders and favoring its chronicity [2,4]. Research to date has ascertained that eating disorders often show significant associations with axis I or II psychiatric disorders—that is, anxiety and depression—in percentages that settle between 80 and 97% of cases [5]. Notwithstanding, the co-occurrence or comorbidity between eating disorders and psychosis is far less investigated, especially in children and adolescents, as findings mostly refer to sporadic adult clinical cases [6–8] which prevent definitive conclusions from being drawn [9]. In a recent study, Solmi and colleagues [9] reported on the significant association between psychotic experiences and eating disorders in a cohort of adolescents, suggesting that psychotic symptoms may be considered as markers of increased risk for more severe psychopathology in late adolescence.

This lack of knowledge is quite surprising. Precocious screening and identification of those who are at risk for full-blown as well as for attenuated forms of psychosis is a priority goal in psychiatric practice with adolescent patients. Additionally, subjects with attenuated forms of psychosis have been found to have a 30% higher probability of developing full-blown psychosis (e.g., schizophrenia) in the following 2 years [10], and they may show reduced response to treatments, especially during adolescence [9,11]. Both a recent large cohort study of more than 6,000 patients [12] and a systematic review of the literature support the hypothesis that eating disorders and psychosis share genetic liability and common phenotypic risk factors. In line with this hypothesis, it is possible to consider eating disorders—and especially anorexia nervosa—as characterized by the intense fear (anxiety) of fattening and delusional beliefs about food, as well as the presence of a distortion of the body image (dysmorphophobia) [6,7].

To sum up, despite clinical evidence suggests a possible overlap between eating disorders and psychosis, to date, the potential association between these two psychiatric entities is not fully defined in adolescents. More specifically, there is no systematic investigation of this association and its clinical implications when psychotic symptoms are present in attenuated forms. The present study aimed at providing a detailed characterization of adolescent patients diagnosed with eating disorders in the absence or presence of psychotic symptoms, taking into consideration a wide set of sociodemographic, psychological, and clinical variables. The specific goals were: (a) to assess the psychological functioning and the severity of the eating disorder in adolescents with or without psychotic symptoms and (b) to explore the associations between neonatal, sociodemographic, clinical, and psychological functioning variables with eating disorders severity in the two groups. To the best of our knowledge, this investigation would provide for the first time in literature an overall picture of the specificity of eating disorders conditions in the absence or presence of psychosis risk.

## Methods

### Participants

Ninety-four adolescent patients diagnosed with eating disorders were consecutively enrolled between May 2013 and October 2019 at the Child Neurology and Psychiatry Unit of the IRCCS Mondino Foundation, Pavia, Italy and at the Child and Adolescent Mental Health, San Gerardo Hospital, Monza, Italy. Inclusion criteria were a diagnosis of anorexia nervosa or other restrictive unspecified eating disorders according to Diagnostic Statistical Manual (DSM-5) and age between 12 and 18 years. Subjects were excluded from the study if they presented at least one of the following exclusion criteria: mental retardation, diagnosis of psychotic disorders prior to the enrollment, neurological pathologies, brain injuries, or other medical conditions associated with psychiatric symptoms. The study received the approval of the participating hospitals. All the procedures are consistent with the principles of the World Medical Association Declaration of Helsinki. All the enrolled patients provided written informed consent to participate in the study.

### Procedures

The access of the patients to the hospital units could be for hospitalization, day hospital, and/or outpatient visit. After providing written informed consent, the patients were interviewed by a trained child psychiatrist. The interview focused on (a) clinical anamnesis and sociodemographic data collection and (b) a pool of questionnaires designed to obtain quantitative measures of specific clinical features: eating disorders severity, global social functioning, associated psychological symptoms, and risk of psychosis.

### Measures

#### Preliminary data

The following sociodemographic and clinical characteristics were obtained: age at enrollment, weight and height at enrollment, parental education level, parental job, parental marital status, eating disorders onset age, previous history of eating disorders and any other neuropsychiatric disorders, quality of social relationships and school adjustment, risky behaviors, menarche, irregular period, presence of elimination conducts, binge-eating and/or dysmorphophobia, psychiatric comorbidities, and any drug treatment. Height and weight at enrollment were used to compute the body mass index (BMI). Parental education level and job were used to compute the socioeconomic status index according to Hollingshead [13].

#### Eating disorders severity

The eating disorders severity was measured according to the Morgan-Russell Outcome Assessment Schedule (MROAS) [14]. This scale was previously used in Italian samples of adolescents with eating disorders [15]. The MROAS is a clinical observational scale to assess the clinical outcome of the eating disorders. It includes 10 items rated on a 4-point Likert scale ranging from 1 (satisfying) to 4 (very unsatisfying). A global outcome score ranging from 10 (good) to 40 (poor) is obtained. This scale was filled in by the child neuropsychiatrist.

#### Global social functioning

The assessment of the global social adjustment of the patients was obtained through the clinician-rated Children’s Global Assessment Scale (C-GAS) [16]. The Italian version has been used in previous samples of adolescents with psychiatric morbidities [17]. The scale is separated into 10-point sections that are headed with a description of the level of global functioning and followed by examples matching the given interval. The final score ranges from 1 (the most impaired level of global functioning) to 100 (the superior level of global functioning).

#### Psychological symptoms associated with eating disorders

The Eating Disorder Inventory (EDI-3) [18] is a 91-item self-reporting questionnaire intended to provide a psychological profile of symptoms related to eating disorders and a quantitative measure of their presence and intensity. The Italian version of the EDI-3 [19] provides six composite scales: Eating Disorder Risk Composite (EDRC), Ineffectiveness Composite (IC), Interpersonal Problems Composite (IPC), Affective Problems Composite (APC), Overcontrol Composite (OC), and General Psychological Maladjustment Composite (GPMC). For each composite scale, higher scores reflect worse symptomatology.

#### Risk of psychosis

The Comprehensive Assessment of At-Risk Mental States (CAARMS) [20,21] is a 27-item semistructured interview that provides a quantitative index of risk for full-blown and attenuated psychosis in psychiatric patients. It is a clinical tool devised also to exclude or confirm the diagnosis of an acute psychotic episode, and its Italian version is validated [22]. The CAARMS gives a comprehensive picture of the patient’s premorbid state; the patient is asked to consider the previous 12 months and the period characterized by the maximal severity of the symptoms as a reference for answering the items. The items assess several psychopathological and functional features grouped into subscales: positive symptoms, cognitive alterations, emotional disturbances, negative symptoms, behavioral changes, somatic-motor changes, and general psychopathology. Each subscale is rated on a 6-point Likert scale that ranges from 0 (absence of symptoms) to 6 (daily present or high-intensity symptoms). A score for attenuated positive psychotic symptoms is obtained by summing up the points scored in the first three subscales. According to the CAARMS, the sample was split into two groups: no psychotic risk (HR−) and psychotic risk (HR+). The PPS group included both subjects with attenuated psychotic symptoms and full-blown psychotic illness.

### Plan of analysis

Among the 94 adolescents included in the study, four (4%) had a diagnosis of full-blown psychosis, and they were removed from the present study. As such, the final sample size for statistical analysis was *N* = 90. The two groups (HR−, HR+) were compared for neonatal, sociodemographic, and clinical characteristics by means of *χ* ^2^ test (categorical variables) and independent-sample *t* test (continuous variables). Independent-sample *t* tests were used to compare the two groups for C-GAS and MROAS scores. A multivariate analysis of variance (MANOVA) was used to compare the EDI-3 dimensions between the two groups. To investigate the differential associations between neonatal, sociodemographic, clinical, and psychological functioning variables with the eating disorders severity, a series of Pearson’s bivariate correlations were carried on the whole sample as well as separately for the two groups. In order to adjust for multiple testing, we used the Benjamini–Hochberg false discovery rate procedure to correct for the number of bivariate correlations tested reporting significant associations, setting an adjusted *q*-value of 0.05. All the analyses have been carried with IBM SPSS 25 setting *p* < 0.05.

#### Results

Descriptive statistics and test statistics for the neonatal, sociodemographic, and clinical variables are reported in Table 1 for the two groups. About 11 subjects were outpatients, 18 were in day hospital, and 65 were hospitalized. The BMI was below the 15th percentile for four patients in the HR− group (27%) and 28 patients in the HR+ group (35%), *χ* ^2^ = 3.25, *p* = 0.354. Higher presence of purging behavior (i.e., elimination conducts) and dysmorphophobia emerged for the HR+ group, compared to HR− counterparts. Moreover, HR+ patients used greater amount of drug treatments, especially antipsychotic medications.

**Table 1.** Descriptive statistics and clinical characteristics of the sample.

	Groups				Group comparison	
	HR− (*N* = 15)		HR+ (*N* = 79)			
	Mean	SD	Mean	SD	*t* test	sig
Age (years)	15.13	2.31	15.53	1.28	0.96	0.338
Eating disorder onset age (years)	14.37	1.82	14.35	1.86	0.04	0.966
Weight (kg)	41.38	7.22	41.39	5.98	0.01	1.000
BMI (kg/m^2^)	16.28	2.26	15.89	2.08	0.65	0.515
Socio-economic status (range: 0–90)	38.00	16.05	31.71	14.16	1.50	0.137

	*N*	%	*N*	%	*χ^2^* test	sig
Parental marital status (separated/divorced)	5	33.3	12	15.2	2.80	0.094
Quality of social relationship (withdrawal or limited)	6	40.0	29	36.7	0.93	0.628
School adjustment (withdrawal or poor)	2	13.3	38	48.1	10.47	0.033
Previous history of eating disorders (yes)	4	26.7	15	19.0	0.46	0.497
Previous history of neuropsychiatric disorders (yes)	3	20.0	22	27.8	0.43	0.512
Risky behaviors (yes)	2	13.3	16	20.3	0.42	0.519
Menarche (no)	2	13.3	7	8.9	0.29	0.589
Period (amenorrhea or irregular)	13	86.7	75	94.9	2.47	0.481
Elimination conducts (yes)	2	13.3	38	48.1	6.23	0.013
Binge-eating (yes)	3	20.0	14	17.7	0.04	0.834
Dysmorphophobia (yes)	12	80.0	74	93.7	3.03	0.082
Main diagnosis (anorexia nervosa)	10	66.7	65	82.3	1.91	0.167
Psychiatric comorbidity (yes)	8	53.3	28	35.4	1.71	0.191
Anti-depressant drug treatment (yes)	5	33.3	25	31.6	0.02	0.898
Anxiolytic drug treatment (yes)	1	6.7	11	13.9	0.60	0.440
Anti-psychotic drug treatment (yes)	1	6.7	37	46.8	8.45	0.004

Abbreviations: BMI, body mass index; HR−, no psychotic risk; HR+, psychotic risk.

**Figure 1.**
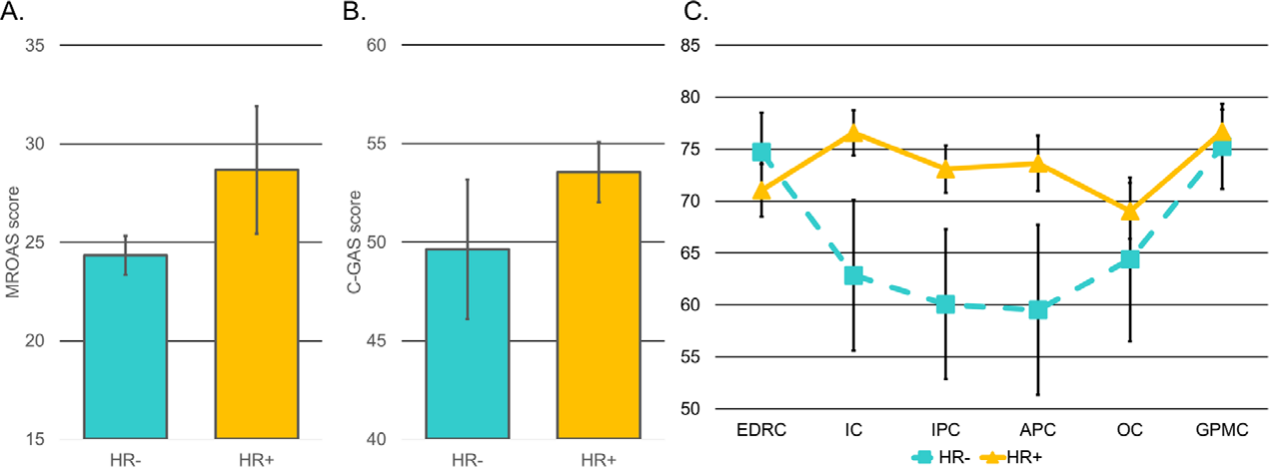
Mean differences in (1A) MROAS, (1B) C-GAS, and (1C) EDI-3 scores between HR− and HR+ eating disorders patients. Abbreviations: APC, Affective Problems Composite; C-GAS, Children’s Global Assessment Scale; EDI-3, Eating Disorder Inventory; EDRC, Eating Disorder Risk Composite; GPMC, General Psychological Maladjustment Composite; HR−, no psychotic risk; HR+, psychotic risk; IC, Ineffectiveness Composite; IPC, Interpersonal Problems Composite; MROAS, Morgan-Russell Outcome Assessment Schedule; OC, Overcontrol Composite. Bars represent standard errors.

The MROAS, C-GAS, and EDI-3 scores for the whole sample and the two groups are reported in Figure 1. Significant differences emerged for the MROAS, *t*(88) = −4.56, *p* < 0.001. The patients in the HR+ group had greater MROAS score compared to the HR− counterpart. No significant differences emerged for the C-GAS score, *t*(88) = −1.24, *p* = 0.219. A multivariate significant effect emerged for the EDI-3, *F*(6,80) = 4.19, *p* = 0.001, *η^2^_p_* = 0.24. Significant group differences emerged for IC, *F*(1,85) = 5.42, *p* = 0.022, *η^2^_p_* = 0.06, IPC, *F*(1,85) = 4.49, *p* = 0.037, *η^2^_p_* = 0.05, and APC, *F*(1,85) = 4.65, *p* = 0.034, *η^2^_p_* = 0.05.

**Figure 2.**
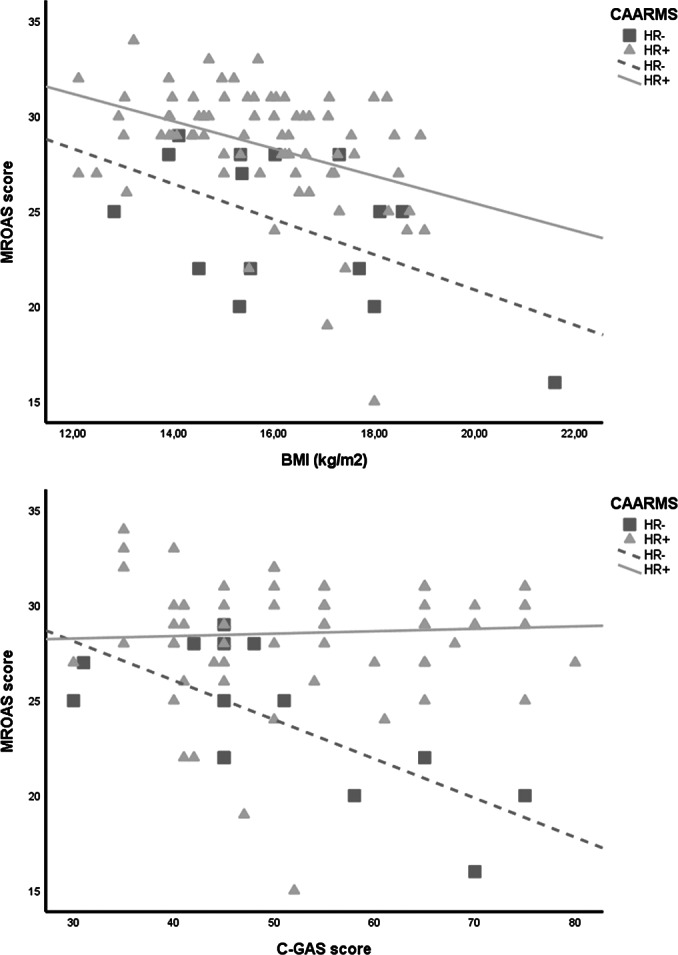
Association of MROAS score with (2A) BMI and (2B) C-GAS score in HR− and HR+ eating disorders patients. Abbreviations: BMI, body mass index; C-GAS, Children’s Global Assessment Scale; HR−, no psychotic risk; HR+, psychotic risk; MROAS, Morgan-Russell Outcome Assessment Schedule.

After correcting for multiple comparison bias, correlational analyses revealed that MROAS score was significantly and negatively associated with BMI in the whole sample, *r* = −0.44, *p* < 0.001, and both in HR−, *r* = −0.55, *p* = 0.035, and HR+ patients, *r* = −0.40, *p* < 0.001. C-GAS score significantly and inversely correlated with MROAS score only in the HR− group, *r* = −0.71, *p* = 0.005 (Figure 2).

#### Discussion

Attenuated psychotic symptoms appeared to be highly frequent in the subjects with eating disorders enrolled in our study: in fact, they were present in 79 out of 94 adolescents (84%). This finding is consistent with previous research suggesting that the comorbidity between psychosis and eating disorders may be at least partially due to overlapping common psychological processes distorting reality perceptions that are common of anorexia nervosa and psychotic symptoms [4]. Moreover, these patients received greater amount of antipsychotic drugs with respect to non-at-risk counterparts. Nonetheless, future research is warranted to investigate whether adolescents with eating disorders and subthreshold psychotic symptoms could mainly benefit from taking antipsychotic drugs with respect to patients who are not at-risk for psychosis.

### Comorbidity between eating disorders and psychotic symptoms

As for the main aims of the study, the findings are reminiscent of a potential psychotic core, concerning body image and weight as well as psychological characteristics. First, we found that impulsive behavior (e.g., purging acts) was much more frequent among at-risk patients. It is relevant to underline that purging behavior in eating disorders is considered as a predictor of poor outcome, associated with delayed time to remission, long duration of illness, greater psychopathology, and higher psychological distress [23,24]. Moreover, impulsivity is a common feature of subjects who are at risk for psychosis [25]. Consistently, we hypothesize that the described impulsiveness of subjects at risk of psychosis, together with a fragility of thought organization, leads to a greater behavioral discontrol, which is observed in terms of greater purging behaviors frequency in these patients.

Second, patients with psychotic symptoms also reported a higher proportion of poor academic achievements and school withdrawal. Research into the cognitive performance and academic achievements of adolescents with eating disorders is limited. Previous research suggests that adolescents with problems in body image and disordered eating behavior are at higher risk for academic interference and lower grade point average [26]. In the same study, the severity of eating disorder symptoms was positively correlated with school maladjustment. Consistently, while academic interference may be a relatively unexamined, yet potentially relevant outcome for individuals who experience eating problems, the present findings suggest that the comorbid presentation with subthreshold psychotic symptoms may further exacerbate the risk of academic failure and school withdrawal.

Third, dysmorphophobia was also much more frequent among patients at risk for psychosis, despite this difference only reached marginal significance. This finding is in line with current literature: distortions of body image are among the most common psychotic symptoms reported in association with eating disorders [5,27]. They play an important role in the development, maintenance, and severity of the eating disorder itself [28,29]. These symptoms are described in previous reports on adult population as the most pervasive, the first that occur (about 6 months before the diagnosis) and the most difficult to treat [27,30]. Nonetheless, replication in larger cohorts is needed to further confirm the significance of this finding.

### Factors influencing the link between eating disorders severity in patients with and without psychotic symptoms

Higher eating disorders’ severity emerged for patients at risk for psychosis, especially in presence of low BMI. This finding further suggests the presence of a link between the severity of the eating disorder and the risk of psychotic subthreshold features. To the best of our knowledge, our finding is the first report on this association in adolescents. A potential and clinically relevant speculation regards the hypothesis that patients with eating disorders who also are at risk for psychosis may present greater thought disorganization which in turn might limit the effectiveness of therapeutic interventions [31].

Notably, a significant association between the severity and global social functioning emerged in patients without risk of psychosis but not in at-risk subjects. We speculate that in the presence of subthreshold psychotic symptomatology, the overall psychological functioning—including relational, social, and school adaptation—is probably much more affected by the frequency and the intensity of psychotic symptoms rather than by features specific of eating disorders [32].

Specific psychological symptoms emerged associated with risk of psychosis in patients with eating disorders. This symptom constellation was characterized by more severe self-perceived ineffectiveness, interpersonal, and affective problems. As such, it could be hypothesized that the attenuated psychotic symptoms observed in a wide proportion of patients with eating disorders may be a proxy of the psychopathological severity and complexity of the eating disorder (e.g., multiple comorbidities, worse overall functioning).

Partially surprising, the global psychological functioning did not differ between the two groups. It should be highlighted that patients with full-blown psychosis, a condition that usually correlates with poor global psychological functioning, were not included in the sample because of limited sample size. At-risk patients presented only subthreshold psychotic symptomatology, identifying a condition that does not necessarily determine a poor global functioning, especially during the early stages of the disorder. This may partially explain the absence of significant difference between the two groups.

### Limitations and future directions

There are several limitations that should be considered when interpreting our results. First, sample size did not allow us to compare subthreshold and full-blown psychosis subjects. Notably, full-blown psychosis is described to occur sporadically in adult patients with eating disorders [33], whereas there is no systematic report of its prevalence in adolescence. Second, this study aimed at describing the overlapping and comorbid presentation of eating disorders and psychotic symptoms in adolescents; as such, no data are reported on follow-up and the outcome of the healthcare journey of these patients, in particular about the transition rate risk to full-blown psychosis. As previous studies in other populations reported 30% transition rate to full-blown psychosis within 2 years from the initial assessment [10], future research should report the transition rate risk in adolescent patients with eating disorders and subthreshold psychotic symptoms. Third, in the present sample, we were not able to collect complete data about the effects of different treatment options, including pharmacotherapy, on the developmental outcomes of the patients. We suggest that this should be prioritized in future research in the field.

## Conclusions

The present study described a multidimensional psychotic core that can have relevant implications for clinical practice with adolescents with eating disorders who are at risk for psychosis. First, the availability of reliable and valid markers of risk can further increase our capacity to detect early emergence of psychosis in adolescents with eating disorders. This study highlights specific dimensions of psychological functioning that can be easily detected by the clinician during anamnestic interviews. More specifically, the present study confirmed that the CAARMS should be regarded as a tool of relevant clinical utility for identifying prodromal psychotic symptoms even in adolescents with eating disorders [34]. We strongly suggest to include CAARMS interview into the ordinary diagnostic assessment of eating disorders. Second, this study may be considered as a first step forward to identify specific psychological dimensions (i.e., inadequacy feelings, interpersonal, and affective regulation problems) that can be the focus of preventive and early intervention strategies. As future research will corroborate and extend our knowledge of the psychotic core in adolescents with eating disorders, these findings are warranted to crucially support the early characterization of patients’ symptoms and needs, the clinicians’ decision-making, and the development of individualized and tailored treatments.

## Data Availability

Data are available upon reasonable request to the corresponding author.

## Abbreviations

APC: Affective Problems Composite
C-GAS: Children’s Global Assessment Scale
EDI-3: Eating Disorder Inventory
EDRC: Eating Disorder Risk Composite
GPMC: General Psychological Maladjustment Composite
HR−: no psychotic risk
HR+: psychotic risk
IC: Ineffectiveness Composite
IPC: Interpersonal Problems Composite
MROAS: Morgan-Russell Outcome Assessment Schedule
OC: Overcontrol Composite
